# Infant Corpus Callosum Size After Surgery and Critical Care for Long-Gap Esophageal Atresia: Qualitative and Quantitative MRI

**DOI:** 10.1038/s41598-020-63212-3

**Published:** 2020-04-14

**Authors:** Chandler R. L. Mongerson, Camilo Jaimes, David Zurakowski, Russell W. Jennings, Dusica Bajic

**Affiliations:** 1 Department of Anesthesiology, Critical Care and Pain Medicine, Boston Children’s Hospital, 300 Longwood Ave., Boston, MA 02115 USA; 2 Department of Radiology, Division of Neuroradiology, Boston Children’s Hospital, 300 Longwood Ave., Boston, MA 02115 USA; 3 Harvard Medical School, Harvard University, 25 Shattuck St., Boston, MA 02115 USA; 4 Department of Surgery, Boston Children’s Hospital, 300 Longwood Ave., Boston, MA 02115 USA; 5 Esophageal and Airway Treatment Center, Boston Children’s Hospital, 300 Longwood Ave., Boston, MA 02115 USA

**Keywords:** Brain, Predictive markers, Neurodevelopmental disorders, Paediatric neurological disorders

## Abstract

Previous studies in preterm infants report white matter abnormalities of the corpus callosum (CC) as an important predictor of neurodevelopmental outcomes. Our cross-sectional study aimed to describe qualitative and quantitative CC size in critically ill infants *following* surgical and critical care for long-gap esophageal atresia (LGEA) – in comparison to healthy infants – using MRI. Non-sedated brain MRI was acquired for full-term (n = 13) and premature (n = 13) patients following treatment for LGEA, and controls (n = 20) <1 year corrected age. A neuroradiologist performed qualitative evaluation of T1-weighted images. ITK-SNAP was used for linear, 2-D and 3-D manual CC measures and segmentations as part of CC size quantification. Qualitative MRI analysis indicated underdeveloped CC in both patient groups in comparison to controls. We show no group differences in mid-sagittal CC length. Although 2-D results were inconclusive, volumetric analysis showed smaller absolute (F(2,42) = 20.40, p < 0.001) and normalized (F(2,42) = 16.61, p < 0.001) CC volumes following complex perioperative treatment for LGEA in both full-term and premature patients, suggesting delayed or diminished CC growth in comparison to controls, with no difference between patient groups. Future research should look into etiology of described differences, neurodevelopmental outcomes, and role of the CC as an early marker of neurodevelopment in this unique infant population.

## Introduction

Long-gap esophageal atresia (LGEA) is a rare birth defect characterized by a long gap of the esophagus (gap wider than 3 vertebral bodies) that can not be repaired by direct approximation. If left untreated, infants are unable to swallow food and secretions, leading to inadequate growth and infections, respectively. Although the Foker process^1,2^ is considered standard of care for treatment of LGEA, there is a major gap in our understanding of the impact of such complex perioperative critical care on brain development. Evidence suggests that critical illness and prolonged mechanical ventilation in infancy are important risk factors associated with altered brain development^3,4^. Emerging reports also suggest that infants born with noncardiac congenital anomalies undergoing surgery and complex critical care in infancy are at increased risk of brain injury^5,6^ and poor long-term outcomes^7,8^. Similarly, infections in infancy were linked to altered brain development and higher risk of long-term neurological sequelae^9^.

We previously reported that critically ill full-term and premature infants following LGEA repair had previously unrecognized clinically significant MRI findings, as well as quantitative increase of cerebrospinal fluid and globally decreased brain size^6,10^. In the same pilot cohort of full-term and premature patients, we also illustrated qualitative MRI findings implicating abnormal thinning of the corpus callosum (CC)^6^ following completion of complex perioperative critical care for LGEA that included Foker process^1,2^ and prolonged postoperative sedation (≥5 days)^11^. The CC forms the major commissural white matter tract important for integrating and transferring interhemispheric signals spanning sensory, motor, and higher-order cognitive domains^12^, and is formed as early as 18–20 weeks gestational age (GA)^13^. Indeed, impaired callosal thickness has been associated with degree of prematurity^14^. Smaller CC size in former premature infants in comparison to full-term-born infants has been reported at term-equivalent age^15,16^, early childhood^17^, and adolescence^18,19^. However, the impact of Foker process and perioperative critical care on CC size in full-term patients is unknown.

Our aim in present descriptive pilot study was to evaluate both qualitative and quantitative MRI measures of CC size in aforementioned pilot cohort of infants with LGEA^6,10^. We hypothesized that critically ill full-term and premature patients <1 year-old following thoracic surgical and critical care for LGEA – in comparison to healthy full-term infants – have (1) higher incidence of qualitative CC abnormalities (shape, size, and degree of myelination), and (2) smaller absolute and normalized CC size using linear, 2-D, and 3-D measures. Due to the nature of the disease and critical care they undergo, infants born with LGEA also receive prolonged parenteral nutrition. Our secondary outcome measure was total body weight (kg) at the time of MRI scan, since feeding regime with total parenteral nutrition does not offer the same nutritional advantages as breast milk^20,21^.

## Methods

### Study design and participants

This work builds on our previous work^6,10^ approved by Boston Children’s Hospital Institutional Review Board as a ‘no more than minimal risk’ study. Informed written parental consent was obtained prior to subject participation, in accordance with the Declaration of Helsinki and Good Clinical Practice guidelines. Considering that data presented is from the same infant study subjects, previously described methodological approach for (1) recruitment criteria and (2) MRI scanning process^6^ apply here. We analyzed 3 groups: full-term patients, preterm patients, and full-term healthy controls. Recruitment was previously described in detail^6^. A parent or legal-guardian of each subject attested to informed consent for study participation. Patients’ eligibility criteria were: full-term (37 to 42 weeks GA at birth) and moderate-to-late preterm (28 to 36 weeks GA at birth) patients <1 year gestation-corrected age that underwent surgery for Foker process for LGEA repair^1,22^. We selected infants that required prolonged postoperative sedation (≥5 days) associated with development of pharmacological dependence to drugs of sedation^11,23–25^. Representative timeline illustrating sequence of perioperative critical care was presented previously^6,26^. Exclusion criteria were: (1) extreme prematurity (<28 weeks GA); (2) ECMO exposure; (3) cranial ultrasound findings (e.g. ventricular enlargement with or without gray matter and/or ventricular hemorrhage); (4) neurological disease (e.g. seizures); (5) chromosomal abnormalities (e.g. Down syndrome); (6) prenatal drug exposure; and/or (7) MRI incompatible implants.

**Figure 1:**
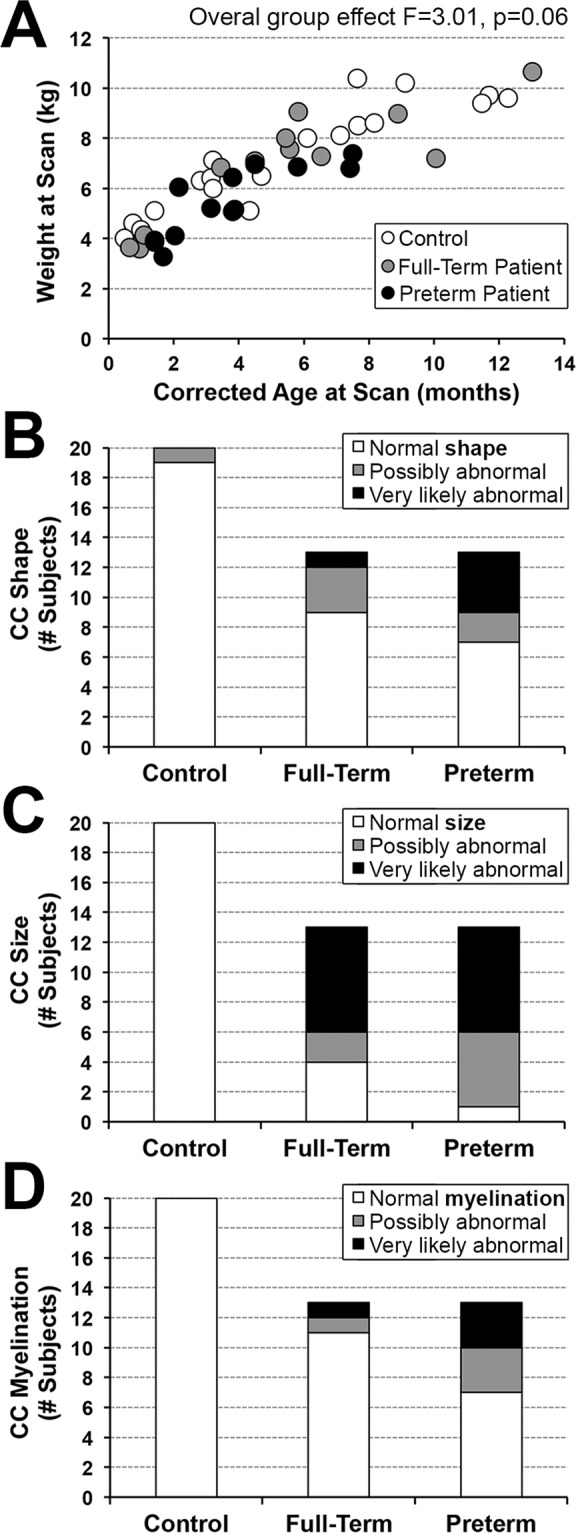
Body Weight (**A**) and Qualitative Analyses of Corpus Callosum (**B**–**D**). Graph A shows individual absolute body weight at scan (kg) for the 3 groups analyzed: (1) full-term controls (n = 20; open circles), (2) full-term patients (n = 13; gray circles), and (3) premature patients (n = 13; black circles). Despite noted trend for group differences, we report no significant differences in body weight at scan between the groups (F(2,42) = 3.01, p = 0.06). **Graphs B–D** summarize results of qualitative MRI analysis of the corpus callosum (CC). Abnormal CC shape (**Graph B**) was either *possible* or *very likely* in 4/13 (31%) full-term patients and 6/13 (46%) preterm patients. In contrast, only 1/20 (5%) controls exhibited possible abnormal CC shape. Incidence of abnormal CC size for age (**Graph C**) was high in both patient groups, with smaller size either *possible* or *very likely* in 9/13 (69%) full-term and 12/13 (92%) preterm patients. Incidence of abnormal CC myelination for age (**Graph D**) was either *possible* or *very likely* in only 2/13 (15%) full-term patients and 6/13 (46%) preterm patients. In contrast, controls showed no evidence of CC abnormalities in age-appropriate size **(C)** or myelination **(D)**.

Healthy full-term infants <1 year old with no prior exposure to surgery, anesthesia or sedation served as a reference baseline for typical CC size and were not age or gender matched. In our original report, we used n = 17 controls for *qualitative* analyses of **T1-weighted** and *quantitative* analyses of **T2-weighted** MRI data^6^. The following changes account for this report: (i) addition of three new control subjects, and (ii) replacement follow-up scans for 2 previously analyzed infants to improve quality of T1-weighted images (now 3.2 months-old control subject and 5.4 months-old full-term patient). Summary of recruitment details and final group characteristics are shown in Table 1. Our secondary outcome measure of individual absolute body weights of subjects at the time of MRI scan is shown in Fig. 1A.

**Table 1:** Recruitment and Group Characteristics.

n	Controls	Full-Term Patients	Premature Patients
***Recruitment Process***			
Considered/(Chart) Reviewed	62	173	108
Eligible (%Reviewed)	59 (95%)	63 (36%)	49 (45%)
Approached (%Eligible)	56 (95%)	40 (63%)	23 (47%)
Consented (%Approached)	22 (39%)	19 (48%)	18 (78%)
Scanned (%Consented)	22 (100%)	13 (68%)	13 (72%)
Included/Analyzed (%Scanned)	20 (91%)	13 (100%)	13 (100%)
***Group Characteristics***			
Sex (male), n (%)	17 (85%)	7 (54%)	8 (62%)
GA at birth (weeks), Mean ± SD	39.3 ± 1.15	38.5 ± 1.1	32.2 ± 2.9
CA at scan (months), Median [range]	4.5 [0.5–12.3]	5.4 [0.7–13.0]	3.8 [1.4–7.5]
Twin births, n (%)	1 (5%)	1 (8%)	2 (15%)
Primary diagnoses			
Isolated LGEA, n (%)	0	3 (23%)	3 (23%)
LGEA with TEF, n (%)	0	5 (38%)	9 (69%)
Other, n (%)	0	5 (38%)	1 (8%)

Table summarizes study recruitment process for the 3 groups (full-term controls, and full-term and preterm patients), as well as demographic and clinical characteristics of all subjects included in analysis of **T1-weighted** images. Numbers for recruitment process are updated since our previous publication^6^ (see Methods). Primary diagnoses included: (1) isolated long-gap esophageal atresia (LGEA), (2) LGEA with tracheo-esophageal fistula (TEF), or (3) other that included LGEA as part of VACTERL association (without cardiac component). Infants diagnosed with VACTERL typically exhibit ≥3 of the characteristic features (viz. **V**ertebral defects; **A**nal atresia; **C**ardiac defects; **T**racheo-**E**sophageal fistula; **R**enal anomalies; **L**imb abnormalities). None of the infants included in analysis were exposed to extracorporeal membrane oxygenation or had any previously known neurological injury or disease. For other exclusion criteria, see Methods. *Abbreviations*: **GA**, gestational age; **CA**, corrected age.

### MRI acquisition

Our MRI scanning protocol was previously described in detail^6,10^. All infants underwent a non-sedated research scan after completion of all perioperative treatment for Foker process using a ‘feed and wrap’ approach^27–30^. Patients were scanned in late evenings or at night using a 3 T TrioTim MRI system equipped with 32-channel receive-only head coil and body-transmission (Siemens Healthcare Inc., USA). Structural **T1-weighted** images were acquired using a MPRAGE sequence (repetition time = 2.52 s; echo time = 1.74 ms; flip angle = 7°; field of view = 192 × 192 mm2; voxel size = 1 × 1 × 1 mm3; 144 sagittal slices). T1 images were collected for all scanned full-term and preterm patients (n = 13/group), and 20/22 (91%) full-term controls (Table 1). Of those 20 controls, only one infant had partial brain coverage that precluded analysis of total brain volume (n = 19 controls) but allowed for quantification of CC metrics. We noted minor ringing artifact due to motion only in 1/20 controls and 1/13 premature patients that did not obscure CC delineation and segmentation.

### Qualitative brain MRI evaluation

The neuroradiologist blinded to treatment groups reviewed T1-weighted scans to identify abnormalities in the CC involving: (i) shape, (ii) size, and (iii) status of myelination for age (Fig. 1B–D). Subjects were divided into three categories based on likelihood of CC finding with respect to each analysis (shape, size, myelination): (1) *normal*, (2) *possibly* abnormal, or (3) *very likely* abnormal CC (e.g. abnormal shape, global or regional thinning, and/or hypomyelination or incomplete myelination for the respective age). Category designations were determined as per the clinical neuroradiologist’s expertise. Myelination scoring was adapted from a study by Plecko *et al*.^31^.

### Quantitative brain MRI analyses

**Figure 2:**
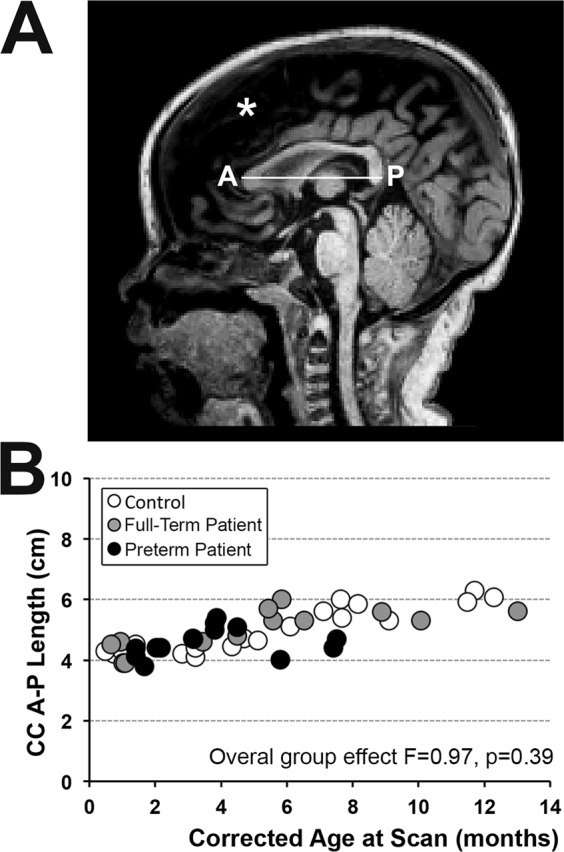
Linear Metrics of Infant Corpus Callosum. Panel A illustrates representative realigned **T1-weighted** brain MRI at mid-sagittal section of a *full-term* patient scanned after perioperative care for long-gap esophageal atresia treatment at 5 months corrected age. It also illustrates linear measurement of total corpus callosum anterior-posterior (A-P) length. Corresponding graph **(B)** shows individual absolute total A-P length (cm) for the 3 groups: (1) full-term controls (n = 20; open circles), (2) full-term patients (n = 13; gray circles), and (3) preterm patients (n = 13; black circles). There were no significant differences in total A-P length between groups (F(2,42) = 0.97, p = 0.39) despite noted increase with age (F(1,42) = 64.86, p < 0.001). Increased presence of extra-axial, interhemispheric fluid in patients (asterisk) necessitated segmentation of intracranial surface area (see Fig. 4A) rather than brain surface area for normalization of corpus callosum (CC) size at this mid-sagittal brain section.

A single blinded rater with neuroanatomical expertise performed data analysis. **T1-weighted** image preprocessing included alignment along the anterior commissure - posterior commissure line using Freeview (v.2.0) to correct for any head tilt during MRI acquisition as illustrated in our previous report (see Fig. 2 in^6^). ITK-SNAP software (v.3.6.0; www.itksnap.org)^32^ was used to measure A-P length (cm) of the CC and to manually segment both CC 2-D surface area (cm^2^) and 3-D volumetric masks (cm^3^). We used a neuroanatomical atlas^33^ and a fiber tract-based atlas of human white matter^34^ as anatomical references for CC measures and segmentation.

#### Linear CC measure

A linear segment running from the most anterior tip of the genu to the most posterior point of the splenium in the mid-sagittal view was used to create an anterior – posterior (A-P) line that represents CC length (Fig. 2A).

#### Surface area (2-D) segmentation

**Figure 3:**
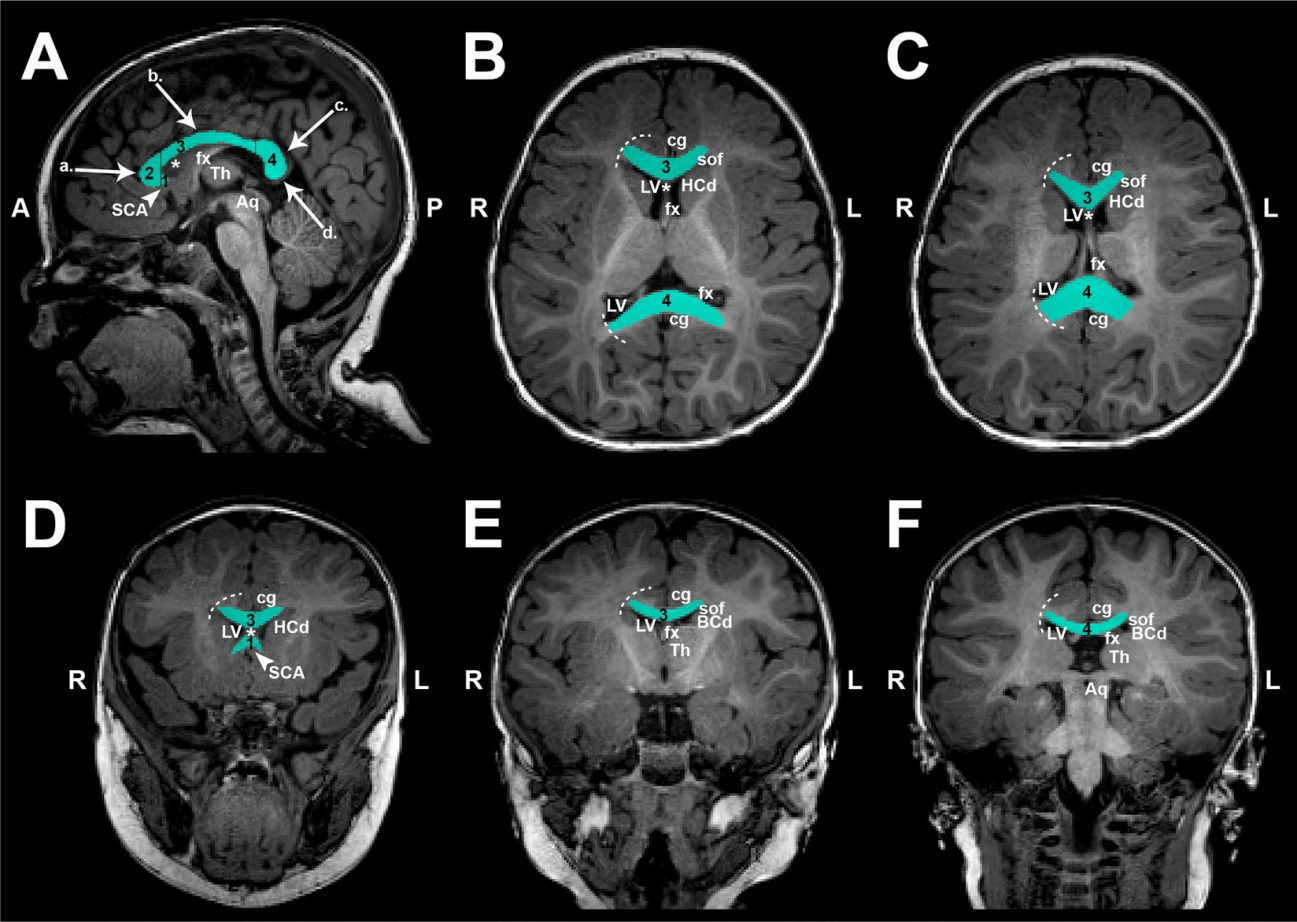
Manual Segmentation of Corpus Callosum. Illustrative tracings of the corpus callosum (CC; cyan) overlaid on T1-weighted sections (gray-scale) of a full-term control scanned at 7.6 months of age. Segmentation of CC included all four main subdivisions: **[1]** rostrum, **[2]** genu, **[3]** body, and **[4]** splenium. As outlined in Panel (A**)** (mid-sagittal section that was used for 2-D surface area analysis), great care was taken to exclude subcallosal area (SCA; arrowhead) and encircling cerebral vasculature including: **[a.]** anterior cerebral arteries, **[b.]** pericallosal arteries, **[c.]** inferior sagittal sinus and pericallosal veins, and **[d.]** great cerebral vein of Galen. Anatomical landmarks important for 3-D volumetric CC segmentation are illustrated in axial (Panels B,C) and coronal sections (Panels D–F). Specifically, lateral outermost edges of the CC were traced as an arching line (white dashed lines) from tip of lateral ventricles (LV) to the in-folding of the cingulate gyrus (cg). Extra attention was given to ensure exclusion of the fornix (fx) columns and crura **(B)** and body **(C)**, as shown in two axial sections moving inferior to superior, respectively. Panels (D–F) show the CC in three coronal sections, moving anterior to posterior: Panel (D), transition from genu **[2]** to rostral body of CC **[3]**; Panel (E), cross-section through mid-body of CC **[3]**; and Panel (F), transition from body **[3]** to splenium **[4]**. Anatomical abbreviations were guided by previous literature^66^. ***Abbreviations***: *, septum pellucidum; **A**, anterior; **Aq**, cerebral aqueduct; **BCd**, body of caudate; **cg**, cingulum; **fx**, fornix; **HCd**, head of caudate; **L**, left; **LV**, lateral ventricles; **P**, posterior; **R**, right; **SCA**, subcallosal area; **sof**, superior occipitofrontal (subcallosal) fasiculus; **Th**, thalamus.

**Figure 4:**
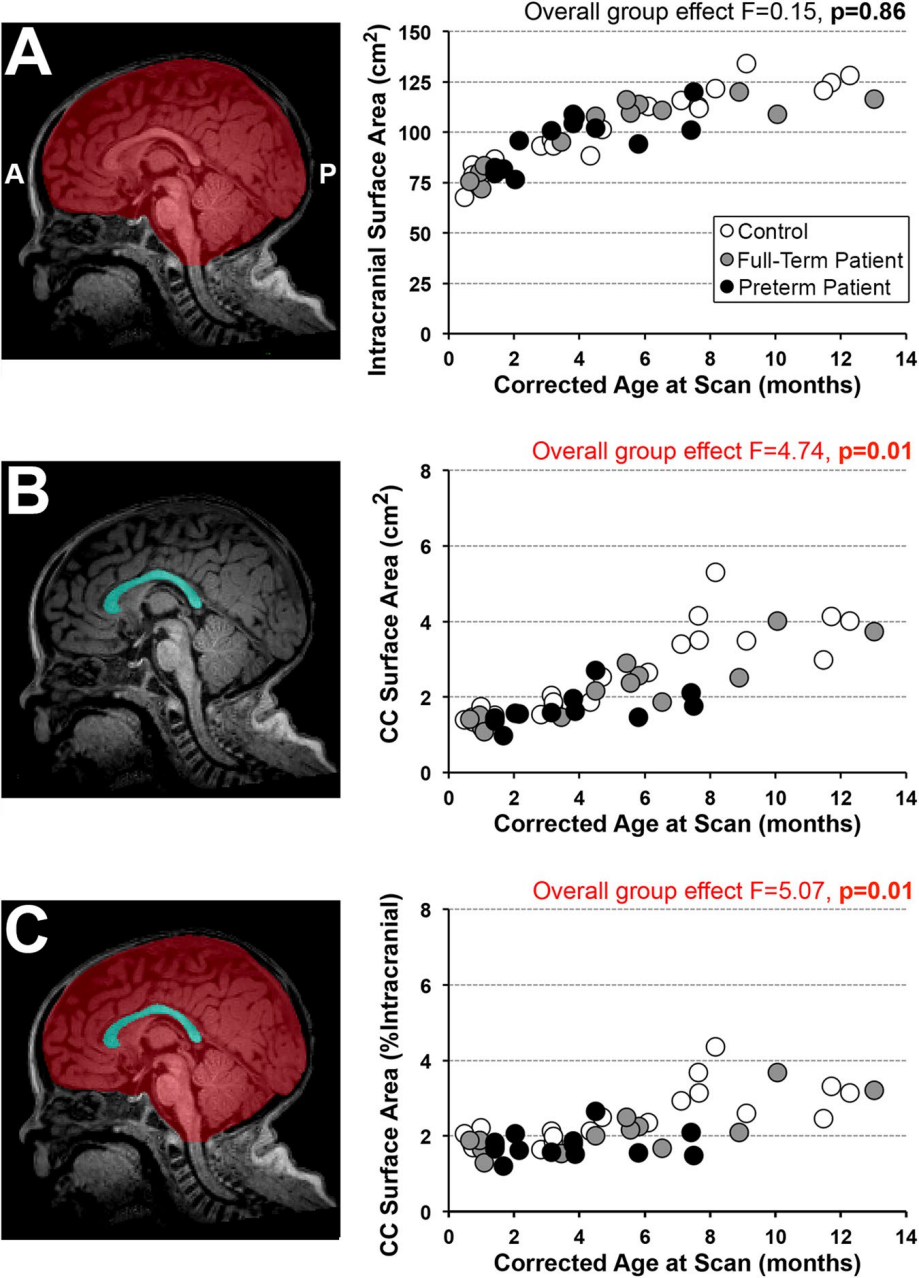
Corpus Callosum Surface Area Analysis. Representative photomicrographs of a T1-weighted mid-sagittal brain section illustrate 2-D surface area segmentations of intracranial space (**A**, red), corpus callosum (CC; **B**, cyan) or both (**C**). Corresponding graphs show quantitative 2-D analysis for the 3 groups: (1) full-term controls (n = 20; open circles), (2) full-term patients (n = 13; gray circles), and (3) premature patients (n = 13; black circles). *Absolute* mid-sagittal intracranial surface area (cm^2^; **graph A**) was not different between groups. In contrast, *absolute* CC surface area (cm^2^; **graph B**) differed among the groups, with significantly smaller CC surface area only between full-term patients and controls (p = 0.005). Likewise, *normalized* CC surface areas (% intracranial space surface area; **graph C**) differed among the groups with significantly smaller normative CC surface area found only between full-term patients and controls (p = 0.004).

A single mid-sagittal section (Figs. 2, 3A and 4) was used to manually trace the surface area of both the CC and intracranial space (Fig. 4). Mid-sagittal surface area of total brain tissue was not evaluated due to variable amounts of extra-axial interhemispheric fluid, particularly in that of patients (Fig. 2A, asterisk). The mid-sagittal section allowed for the best visualization of CC boundaries and was identified by the presence of anterior and posterior commissures, cerebral aqueduct, septum pellucidum, and distinctness of the thalamus. Callosal fibers sharply contrast with neighboring gray matter and cerebrospinal fluid^15,35^ allowing for straightforward manual segmentation of the CC.

#### Volumetric (3-D) segmentation

We manually segmented 3-D CC and performed whole brain extraction, as described below.

*3-D CC Segmentation.* Anatomical landmarks important for 3-D demarcation of CC boundaries are summarized in Fig. 3. This is in line with previously described protocol for CC segmentation in newborns by Yu *et al*.^36^. Great care was taken to exclude encircling cerebral vasculature and neighboring subcallosal area inferiorly (Fig. 3A). Challenging structural distinctions included the cingulum and fornix (columns, body, and crux) due to their close proximity to CC contour throughout its length (Fig. 3B–F). We also extended previously described protocol^36^ by defining lateral boundaries of CC segmentation. Specifically, lateral outermost edges of the CC were bounded by the anterior and posterior corona radiata^34^. Effectively, this was traced as an arching line from tip of lateral ventricles to the in-folding of the cingulate gyrus (Fig. 3B–F; white dashed lines).

*3-D Total Brain Segmentation.* We also performed semi-automated total brain tissue segmentation of T1-weighted images that was necessary for normalized CC volume calculation. It included: (i) Skull-stripping of T1 images by manually tracing whole brain outline (includes ventricular system); and (ii) Partial volume segmentation of cerebrospinal fluid (CSF) using **F**MRIB’s **A**utomated **S**egmentation **T**ool (FAST)^37^. We found that FAST served well in distinguishing between CSF and brain tissue, even beyond the neonatal period. Using tools in **F**MRIB **S**oftware **L**ibrary (FSL; v.5.0), CSF partial volume estimate was (a) thresholded at 99% (eliminating voxels with <99% of their volume comprising CSF), (b) converted to a binary CSF mask, which was then (c) subtracted from the mask of a whole brain outline in order to generate mask of total brain that excludes ventricular system. Brain volume masks underwent additional (d) minor manual editing to draw-in any missing brain tissue.

#### Structural quantification

**Figure 5:**
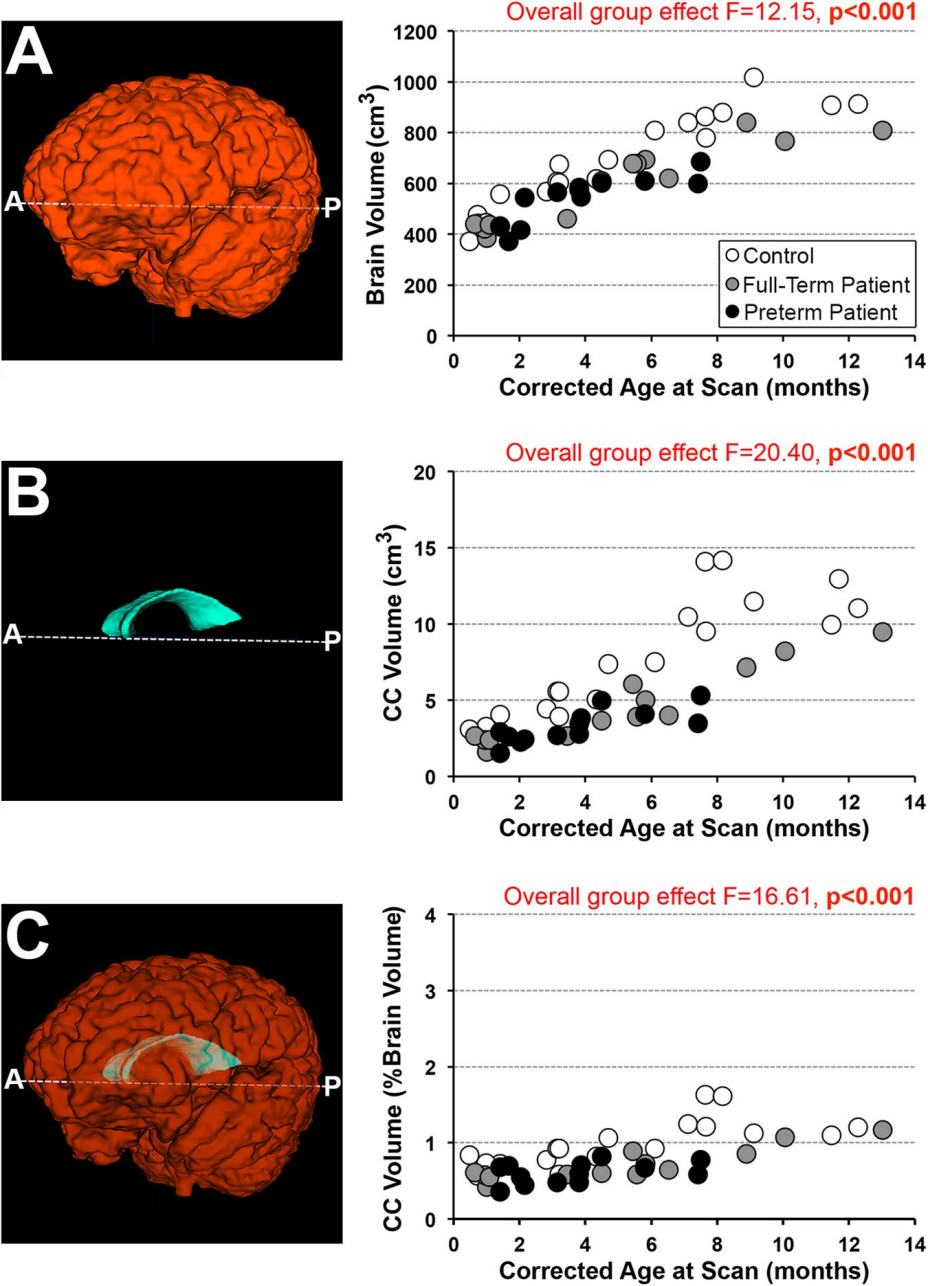
Corpus Callosum Volumetric Analysis. Representative 3-D renderings illustrate segmentations of total brain (**A**, orange), corpus callosum (CC; **B**, cyan) or both (**C**) based on T1-weighted MRI analysis. Corresponding graphs show quantitative 3-D analysis for the 3 groups: (1) full-term controls (n = 20; open circles), (2) full-term patients (n = 13; gray circles), and (3) premature patients (n = 13; black circles). We report differences between groups for all 3 measures: *absolute* brain volume, *absolute* CC volume, and *normalized* CC volume. Specifically, *absolute* brain volume (cm^3^; **graph A**) was smaller in both full-term and premature patients in comparison to controls (p < 0.001), with no difference between patient groups (p = 0.81). Likewise, *absolute* CC volume (cm^3^; **graph B**) was significantly smaller in patients compared to controls (p < 0.001), with no difference between patient groups (p = 0.70). *Normalized* CC volume (%TBV; **graph C**) was smaller in both full-term and preterm patients in comparison to controls (p < 0.001), with no difference between patient groups (p = 0.63). Please note that brain volume used for normalization was not available for 1/20 controls (infant scanned at 11.7 months-old) due to incomplete brain coverage, which accounts for discrepancy in a single data point between the graphs.

Absolute values of 2-D segmentations (intracranial and CC surface area in cm^2^; Fig. 4A,B, respectively) and 3-D segmentations (brain and CC volume in cm^3^; Fig. 5A,B, respectively) were obtained using the ITK-SNAP volume estimation tool. In addition, we calculated normalized values for CC surface area (% intracranial surface area; Fig. 4C) and CC volume (% brain volume; Fig. 5C). Normalization using total brain tissue is appropriate for understanding how CC size changes with respect to the brain as a whole^38^.

### Statistical analysis

As this was a pilot study and no prior information was available regarding brain volumes in the selected cohort of infants with LGEA, a convenience sample size of 13 patients/group was chosen, based on the anticipated number of eligible infants at our institution and an estimated 50% enrollment rate. Statistical analyses were performed using the Statistical Package for the Social Sciences (SPSS, v.23.0; IBM Corporation, Armonk, NY). Normal distribution of all continuous variables was confirmed using the Shapiro-Wilk test. Comparison of volumes between the three groups was assessed using a general linear model (GLM) univariate analysis with corrected age at scan as a covariate and Bonferroni adjusted p values. The interaction (viz. test of parallelism) was reported when significant. Statistical significance was assessed at the α < 0.05.

## Results

T1-weighted images allowed for qualitative and quantitative comparisons of CC size between full-term and premature patients (n = 13/group), and full-term controls (n = 20; Table 1). Total body weight of all subjects in the analysis is illustrated in Fig. 1A. Despite noted trend for group differences, we report increased absolute body weight with advancing age at scan (F(1,42) = 174.65, p < 0.001) with no differences between groups (F(2,42) = 3.01, p = 0.06).

### Qualitative corpus callosum evaluation

The neuroradiologist performed qualitative evaluation of CC, which demonstrated a consistent pattern of either *possible* or *very likely* abnormal shape (Fig. 1B), overall smaller CC size for age (Fig. 1C), and either hypomyelination or incomplete CC myelination for age (Fig. 1D) in both full-term and premature patients with no previously known neurological concerns. Such striking qualitative findings of smaller CC size for age (Fig. 1C) in majority of patients with LGEA fueled quantitative analyses presented below.

### Linear and 2-D MRI quantification

Mid-sagittal length of the CC as measured by linear anterior-posterior (A-P) distance along its longest dimension (Fig. 2A) increased with advancing age (F(1,42) = 64.86, p < 0.001) and showed no significant differences between groups (F(2,42) = 0.97, p = 0.39; Fig. 2B). All 2-D measures significantly increased with advancing age irrespective of group status (Fig. 4): *absolute* intracranial surface area (cm^2^; F(1,42) = 133.41, p < 0.001), *absolute* CC surface area (cm^2^; F(1,42) = 94.15, p < 0.001), and *normalized* CC (% intracranial surface area; F(1,42) = 38.08, p < 0.001). Absolute intracranial surface area did not differ between groups (F(2,42) = 0.15, p = 0.86; Fig. 4A), suggesting head size at scan followed a similar trajectory over time for all. In contrast, group status independently predicted CC size at scan. Specifically, *absolute* CC surface area showed significant group differences (F(2,42) = 4.74, p = 0.01; Fig. 4B) only between full-term patients and controls (p = 0.005), but not preterm patients and controls (p = 0.05) or between patient groups (p = 0.37) which could be attributed to higher variability of data in premature patients and lack of data points for premature infants older than 8 months. Likewise, *normalized* CC surface areas (% intracranial surface area) showed significant group differences (F(2,42) = 5.07, p = 0.01; Fig. 4C) between full-term patients and controls (p = 0.004), but not between preterm patients and controls (p = 0.06) or between patient groups (p = 0.28).

### 3-D MRI quantification

All 3-D measures significantly increased with advancing age irrespective of group status (Fig. 5): *absolute* total brain (cm^3^; F(1,41) = 224.02, p < 0.001) and CC volumes (cm^3^; F(1,42) = 130.29, p < 0.001), and *normalized* CC volume (% total brain volume; F(1,41) = 49.80, p < 0.001). Absolute total brain volume was significantly smaller (F(2,41) = 12.15, p < 0.001; Fig. 5A) in both full-term and premature patients in comparison to controls (both p < 0.001), with no difference between patient groups (p = 0.81). Likewise, *absolute* CC volume was significantly smaller (F(2,42) = 20.40, p < 0.001; Fig. 5B) in both full-term and premature patients in comparison to controls (both p < 0.001), with no difference between patient groups (p = 0.70). Furthermore, significant interaction between age at scan and group status for *absolute* CC volume (F(2,40) = 4.34, p = 0.02; Fig. 5B) suggests possible altered growth trajectories between groups with advancing age. *Normalized* CC volumetric analysis (% total brain volume) showed disproportionately smaller CC size (F(2,41) = 16.61, p < 0.001; Fig. 5C) in both full-term and preterm patients in comparison to controls (both p < 0.001), with no difference between patient groups (p = 0.63).

## Discussion

Building on our previous report of incidental CC findings (see Fig. 5 in^6^), our current descriptive pilot study shows qualitatively and quantitatively smaller absolute CC volume in both full-term and premature infants following complex LGEA treatment. Disproportionately smaller normalized CC volume (relative to total brain tissue) in both patient groups, points to CC as a potential early marker of neurodevelopment. These findings could possibly implicate subtle cortical neuronal and/or astrocytic vulnerability in the setting of LGEA treatment, not previously seen by analysis of gross forebrain structure as a whole^10^.

This study demonstrates qualitatively smaller CC size and increased incidence of possible abnormal shape and hypomyelination or incomplete myelination in both full-term and premature patients (Fig. 1). These results implicate (1) pre-existing (in-utero) brain abnormalities and/or (2) risk of atypical CC development for full-term and premature infants in the setting of complex perioperative critical care for LGEA (postnatal and/or peri-surgery). It has been well established that premature birth is associated with altered white matter development^39^ including that of the CC^40^. Interestingly, a recent study by Stolwijk *et al*.^5^ reported a preponderance of punctate white matter lesions in both full-term and preterm patients following neonatal surgery for major noncardiac congenital anomalies. Authors reported that, in addition to prematurity, the type of congenital anomaly (e.g. including those with esophageal atresia) predicted presence of white matter lesions. Such findings might not be unique to infants with esophageal atresia, since it is known that neonates born with congenital heart defects are at risk of altered CC size pre-surgery, with such disparities intensifying following cardiac surgery and critical care^41^. Additionally, altered cerebral brain perfusion has been implicated in abnormal white matter findings in patients following surgery for congenital diaphragmatic hernia^42^. Whether intrinsic qualitative CC findings are present at birth in our population of infants with LGEA (pre-Foker) remains to be determined.

To our knowledge, this is the first study to use quantitative brain MRI to assess CC size in critically ill full-term and premature infants after complex perioperative care for LGEA repair. In accordance with our previous linear and volumetric findings of intracranial volume (as a correlate of head circumference)^6^, we report larger absolute mid-sagittal intracranial surface area with advancing age without significant group differences, suggesting similar head growth between groups with age (Fig. 4A). Interestingly, length of CC in our pilot cohort does not show group differences (Fig. 2B). Although both absolute and normalized CC surface area (Fig. 4B,C) increased with age for all groups, 2-D measures of CC size were significantly smaller in only full-term patients compared to controls. Literature of premature infants at term-equivalent age^15^ and in adolescence^35,43^ showed negative correlations between absolute surface area of the CC and gestational age. Contrary to our hypothesis, trend towards smaller absolute or normalized CC surface areas in preterm patients compared to controls did not reach significance. Although previous structural MRI studies in infants established feasibility of manual cross-sectional CC surface area analysis^15,44–47^, it is possible 2-D surface area analysis (proxy of CC thickness) requires higher statistical power that is not met in our cohort.

Regarding the 3-D analysis, in accordance with our previous study (using T2-weighted volumetric analysis)^6^, current T1-weighted volumetric analysis also demonstrated smaller total brain size in both full-term and premature patients in comparison to controls (Fig. 5A). Although some patients had VACTERL association (Table 1), none had concomitant hydrocephalus (viz. VACTERL-H)^48,49^, known to be of autosomal recessive or X-linked recessive inheritance^49–52^. Similarly, none of the subjects included in analysis had cardiac involvement (less severe cases). Following exclusion of extremely premature infants (born <28 weeks GA), we report smaller absolute CC volumes in both full-term and premature patients following LGEA repair in comparison to controls (Fig. 5B,C). Since this is an observational pilot study, etiology associated with presented 3D findings is not known. Whether disparities in 3-D CC measures between patients and controls reflect pre-existing (prenatal) and/or delayed (postnatal; peri-surgery) CC maturation remains unclear and awaits future studies that should evaluate growth in contrast to size as described in this study. Our most recent preliminary data published as a small case report series^53^ are in support of possible double-hit etiology of brain injury (pre-Foker and peri-Foker process) in both premature and full-term infants with LGEA. Both the degree of prematurity^54^ and extremely low birth weight^55^ have predicted smaller absolute CC volumes at term-equivalent age. Furthermore, CC volume at term-equivalent age^54^ was predictive of CC size at 7-years of age^44^, suggesting early altered CC growth patterns may persist into childhood. We also report decreased normalized CC volumes in both full-term and premature patients scanned after complex perioperative LGEA treatment. This interesting finding is in contrast to our recent T2-weighted MRI study report^10^, which showed global decrease of gross brain, forebrain, and deep gray matter (viz. thalamus and basal ganglia) volumes. It still remains to be investigated if decreased normalized CC size is paralleled with subtle cortical gray/white matter changes. Follow-up multimodal MRI studies in this clinical population are warranted to assess sub-regional patterns of CC size and growth and its homotopical^56^ sub-regional properties of the cortex. Given its pivotal role in facilitating cross-talk between cerebral hemispheres, aberrant CC development or injury may translate to altered motor and cognitive functional circuitry later in life^57–60^. Notably, former very premature neonates with white matter injury in the CC were shown to exhibit altered intrinsic functional connectivity between thalamic and sensorimotor networks^61^. Future studies of the CC should include complementary diffusion tensor imaging (DTI) analyses such as regional white matter measurements of fractional anisotropy and mean diffusivity as indicators of white matter microstructural integrity in the setting of perioperative critical care in infancy.

To date, investigations into CC size as an early marker of later neurodevelopmental outcomes are limited, largely confined to premature infants, and inconclusive in terms of contradictory findings. In one study of very preterm infants, there was no association between CC size and neurodevelopmental function at 2-year follow-up^54^. In contrast, several recent studies in premature infants reported CC size at term-equivalent age as an important predictor of cognitive outcomes in early childhood^44,62,63^. Other studies showed that reduced CC growth predicts motor performance at 2 years of age^64^ and late childhood^65^. Qualitative thinning of the CC and hypomyelination in very preterm infants at term-equivalent age was associated with IQ and language formation at 4 to 6 years of age^63^. In contrast, a recent study by Thompson *et al*.^44^ reported that smaller total CC surface area in former very preterm children at 7 years of age was associated with higher intelligence and reading skills, but not language and visual function. Given the limited number of studies investigating neurodevelopmental implications of CC growth and varying cohort characteristics and age ranges therein, more research is needed to understand the long-term functional significance of altered CC size of infants with LGEA.

Several limitations should be considered when interpreting results of presented observational pilot study. **(1) Pre-Existing Findings:** None of the recruited infants had any previously known brain abnormalities or neurologic disease. However, since no pre-treatment MRI scans were available, we could not rule out the possibility of inherently altered CC structure. Our preliminary findings implicate possible pre-existing (pre-Foker) CC findings^53^, similar to those previously reported for infants undergoing cardiac surgery and congenital diaphragmatic hernia^41^. **(2) Study Groups:** We lack a true control group since (1) there is no alternate treatment for LGEA that does not involve surgery, (2) there are a limited number of infants with prolonged sedation (without surgery)^11^, or (3) otherwise healthy preterm infants that did not require medical care. Therefore, premature infants with LGEA served as a positive control, while otherwise healthy full-term controls served as negative control in this pilot study. **(3) Study Size:** We had a limited number of subjects scanned at older time points (>8 months of age), particularly in that of the preterm patient group (see Table 1). Age-related changes in CC size were accounted for during statistical analysis by using a general linear model with gestation-corrected age at scan as a covariate. **(4) Timing of the Brain MRI:** Subjects in our one-time cross-sectional study were scanned at a wide range of corrected ages, introducing a potential bias. Future analyses should include uniform age range distribution and additional time points for brain MRI scans (e.g. pre- and post-Foker treatment). **(5) Sex Differences:** Unlike the balanced sex distributions in patient groups, the control group consisted of mostly males (17/20 (85%)). It remains unclear whether sex is a determinant of CC size. One study in a large cohort of neonates reported no sex differences in total cross-sectional area of the CC or its sub-regions^47^. A more recent study of CC size from birth to young adulthood also found no sex differences in *absolute* total CC size, but demonstrated significant female > male trend once normalized to whole brain tissue^45^. Given the limited reports on CC size in infancy, and varying age ranges of cohorts therein, future investigations are needed to establish whether sex-dependent growth patterns exist and to elucidate the precise mechanisms underlying possible postnatal sex differences of CC size.

## Conclusions

This study provides evidence that both full-term and premature infants following LGEA repair are at risk of smaller *absolute* CC size. Equally important, disproportionately smaller *normalized* CC volume (relative to total brain) in both patient groups points to potential heightened vulnerability of this brain structure. In the light of the descriptive and hypothesis-generating nature of this study, future research should look into exact causative factors/mechanisms underlying observed differences including possible pre-existing (prenatal and/or pre-Foker) brain abnormalities, as well as neurodevelopmental outcomes of infants with LGEA and role of the CC as an early marker of neurodevelopment.
